# Leveraging Public Data: Changes in Local Economic Distress and Drug Overdose Deaths at the County Level, 2000–2019

**DOI:** 10.3389/ijph.2025.1607991

**Published:** 2025-02-21

**Authors:** Shaddy K. Saba, Juan M. Lavista Ferres, William B. Weeks

**Affiliations:** ^1^ Silver School of Social Work, New York University, New York City, NY, United States; ^2^ Microsoft, Redmond, WA, United States

**Keywords:** distressed communities index, economic distress, area-level, drug overdose, deaths of despair

## Introduction

Indices of local economic distress, including local-area unemployment, poverty, low income, and low education, have been linked with lower healthcare quality, higher care costs, and poorer health [1]. There exist vast county-level disparities in economic conditions across the U.S., and counties with greater economic distress demonstrate inequities in health and social determinants of health [2]. Improvements in local economic conditions predict better health including reduced mortality [3] and improved cardiovascular outcomes [4].

The early 2000s saw a reduction in life expectancy, driven by an increase in deaths by drug overdose, suicide, and chronic liver disease [5]. These have been termed “deaths of despair;” while overdose deaths during the early 2000s’ opioid epidemic are attributed to complex factors including overprescribing, there are also likely socioeconomic determinants including distressed local economic conditions [5].

We leveraged two public data sources [the Centers for Disease Control and Prevention’s Wide-ranging Online Data for Epidemiologic Research (CDC WONDER; wonder.cdc.gov) database and the Economic Innovation Group’s Distressed Communities Index (DCI; eig.org/dci-fact-sheet)] to examine whether local economic distress and changes in distress were associated local drug overdose. This approach highlights how public data can be used to explore critical public health questions.

## Methods

This study was a secondary data analysis. From CDC WONDER, we obtained U.S. county-level age-adjusted rates of drug overdose deaths per 100,000 people for 2000, 2010, and 2019. Overdose deaths from any psychoactive substance not determined to be a homicide or suicide were included. Counties with less than 20 deaths were excluded because of privacy concerns.

We obtained county-level DCI scores for 2000, 2010, and 2019. DCI scores are constructed by the Economic Innovation Group from seven US Census measures of economic activity, including unemployment, poverty, education, median income, housing vacancy, changes in employment, and changes in business establishments. DCI scores range from 0 (lowest) to 100 (highest economic distress). In contrast to individual indicators of economic distress, this composite measure was designed to be comprehensive and normalized, and it has been linked with mortality outcomes across several domains including due to surgeries and firearms [6, 7].

For cross-sectional analyses, we grouped counties into DCI quintiles and used population-weighted ANOVA to compare overdose deaths between groups, in line with prior work [1, 8]. For longitudinal analyses, we identified counties with overdose death data at both 2000 and 2019 with a DCI score increase (n = 13) or decrease (n = 31) of greater than 10 points, and those that did not experience changes (n = 109). As DCI scores are percentile scores reflecting the distribution of distress across all US counties, a 10-point change represents a meaningful shift in relative economic distress at the county level while also providing large enough sample bins for our analyses. We used population-weighted, repeated measures ANOVA to compare percent change in overdose death rates between DCI change groups. We used Stata version 16 for analyses.

Data were from public sources; IRB approval was not required.

## Results

As deaths generally increased during the study period, the number of counties that were included in the sample (with greater than 20 deaths) increased from 2000 to 2019. In 2000, 153 counties representing 136,972,970 individuals were included; the average age adjusted death rate across all counties was 7.46 (SD = 4.52). In 2010, 367 counties representing 203,625,595 individuals were included; the age adjusted death rate was 15.86 (SD = 13.06). In 2019, 570 counties representing 251,637,046 individuals were included; the age adjusted death rate was 27.91 (SD = 16.82).

In cross sectional analyses, age-adjusted overdose death rates were consistently progressively higher in more economically distressed quintile groups, with only one exception when comparing quintiles 3 and 4 in 2019; there were significant differences between groups (F = 2.91, *p* = 0.024 for 2000; F = 10.94, *p* < 0.001 for 2010; F = 2019, *p* < 0.001 for 2019). Table 1 shows mean overdose death rates and ANOVA results for DCI quintile groups in each year. Death rates increased from 2000 to 2019, as did the number of significant differences between DCI quintile groups per *post hoc* Tukey tests.

**TABLE 1:** Age-adjusted death rates means and comparisons between Distressed Communities Index (DCI) quintile groups in 2000, 2010, and 2019 (United States, 2024).

DCI quintile group	DCI quintile cut point	Death rate mean (SD) per 100,000	Number of counties	Estimated total population	ANOVA		Tukey test significant differences
					F-test	P-value	
2000							
1	6.58	5.0 (2.0)	31	23,739,833	2.91	0.024	5*
2	16.6	6.7 (2.4)	30	27,146,477			
3	31.1	7.7 (2.8)	31	20,780,834			
4	44.3	8.1 (4.2)	30	32,200,716			
5	100	9.9 (7.5)	31	33,105,110			1*
2010							
1	10.5	10.2 (4.4)	74	41,376,490	10.94	<0.001	4**, 5***
2	25.9	12.8 (6.7)	73	42,023,082			5***
3	43.2	14.1 (5.6)	73	48,506,446			5***
4	59.6	15.4 (8.7)	73	45,785,241			1**, 5*
5	100	26.7 (22.8)	74	25,934,336			1***, 2***, 3***, 4*
2019							
1	7.7	19.2 (10.4)	114	55,599,229	23.26	<0.001	2**, 3***, 5***
2	17.5	24.2 (12.2)	114	59,279,350			1**, 5***
3	31.6	27.9 (12.8)	115	45,965,780			1***, 4*, 5***
4	55.6	27.6 (12.2)	113	65,161,116			3*, 5***
5	100	40.6 (24.7)	114	25,631,571			1***, 2***, 3***, 4***

***p < 0.001; **p < 0.01; *p < 0.05 for Tukey multiple comparison test significant differences.

Note. DCI quintile groups are ranked from high to low, with 1 being least distressed counties and 5 being most distressed counties. Death rate is average of age-adjusted number of overdose deaths per 100,000 people within counties in each DCI quintile group. ANOVA test includes adjustment for county size.

**FIGURE 1:**
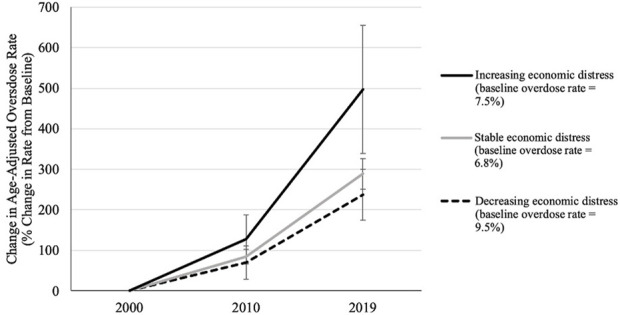
Mean and standard deviation of percent change in age-adjusted overdose death rate by Distressed Communities Index (DCI) change category (United States, 2024). Note. Between 2000 and 2019, “decreasing economic distress” counties’ DCI scores decreased by at least 10 points (Mean DCI change = −18.25; SD = 8.26); “increasing economic distress” counties’ DCI scores increased by at least 10 (Mean DCI change = 17.48; SD = 6.21); and “stable economic distress” counties’ DCI scores changed by less than 10 points (Mean DCI change = −0.78; SD = 4.31). Baseline percent values in the legend indicate raw age-adjusted death rate in 2000 in each group.

While all DCI change categories evidenced increases in overdose deaths from 2000 to 2019, there was a significant time by category interaction (F = 4.70, *p* < 0.001). Counties that became more economically distressed (a DCI increase of greater than 10) experienced a five-fold increase in age-adjusted overdose deaths. In contrast, counties with unchanging levels of economic distress experienced a 2.9-fold increase, and counties becoming less economically distressed (a DCI decrease of greater than 10) saw a 2.4-fold increase in overdose deaths. Figure 1 depicts percent change in overdose death rate for counties with and without DCI score changes.

As a sensitivity check, we re-ran analyses using continuous DCI scores rather than quintile groupings and included county-level race/ethnicity as a covariate. Results mirrored the original analyses (see Supplementary Material).

## Discussion

Worse economic distress was associated with higher drug overdose deaths at the county level between 2000 and 2019. While overdose deaths increased generally over time, counties with worsening distress experienced greater increases in overdose deaths than counties with stable economic distress; improvements in economic distress appeared protective.

Our study aligns with another reporting links between economic distress and deaths of despair [9]. We extend this work by linking a validated measure of county economic distress (the DCI) with a specific outcome (drug overdose deaths). Results suggest local economic policy might impact population health including drug overdose [8].

Our study has several limitations. We only had data on overdose deaths for larger counties, limiting generalizability to smaller counties. Although our quasi-experimental approach strengthens the case for causality, we could not directly evaluate the relationship between local economic conditions and individual-level health outcomes. Our analysis include the pre-COVID 2000–2019 period; results might differ in other periods.

### Conclusion

This study exemplifies how leveraging and combining publicly available data can advance public health knowledge. We demonstrate a strong association between local economic distress (and changes in distress) and drug overdose. We highlight the potential health returns of local economic investment particularly in reducing overdose. Future research should consider whether specific economic policies can address overdose.
