# Supplementary material for: Leveraging Public Data: Changes in Local Economic Distress and Drug Overdose Deaths at the County Level, 2000–2019
Source: Int J Public Health. 2025 Feb 21;70:1607991. doi: 10.3389/ijph.2025.1607991 (PMC11884982; doi:10.3389/ijph.2025.1607991)
Supplement: Supplementary file 1 [file DataSheet1.PDF]

Supplemental Materials

As a sensitivity check, we conducted our analyses using continuous DCI scores rather than DCI quintile or change groupings. In these analyses, we included county proportion of non-Hispanic White individuals as a covariate. Substantive results mirrored the original analyses. For cross sectional analyses for 2000, 2010, and 2019, we ran separate population-weighted linear regression models with data from each year. Continuous DCI scores were significantly, positively associated with age-adjusted drug overdose deaths at the county level in 2000 (b = 0.12, $p < .001$), 2010 (b = 0.16, $p < .001$), and 2019 (b = 32.69, $p < .001$) while accounting for county-level race/ethnicity.

For longitudinal analyses of change in county-level age-adjusted overdose deaths from 2000-2019 and the effect of changing DCI scores, we ran a multi-level regression model. Years were nested by county, allowing us to model and account for the linear trend (change) in county-level overdose deaths. This model included continuous change in DCI over the study period, year (where 2000 was scaled to 0, as it was the start of the study period), and the interaction between change in DCI and year as predictors of overdose deaths. County proportion of non-Hispanic White individuals was also included as a covariate. The interaction between change in DCI and year was significant and positive (b = .09, $p <$ .035), suggesting that changes in the continuous DCI variable magnified the effect of year on area-level overdose deaths between 2000-2019.

The Figure below depicts predicted values for age-adjusted overdose rates, and the influence of the interaction between continuous change in DCI scores and year during the study period, based on our multi-level regression results. Predicted values for +10, 0, and -10 DCI are plotted. As depicted in the Figure, as year increases, age-adjusted overdose rate increases. A DCI score decrease during the study period is predicted to *attenuate* the increase in overdose deaths over time (a less steep slope); whereas a DCI score increase during the study period is predicted to *magnify* the increase in overdose deaths over time (a steeper slope).

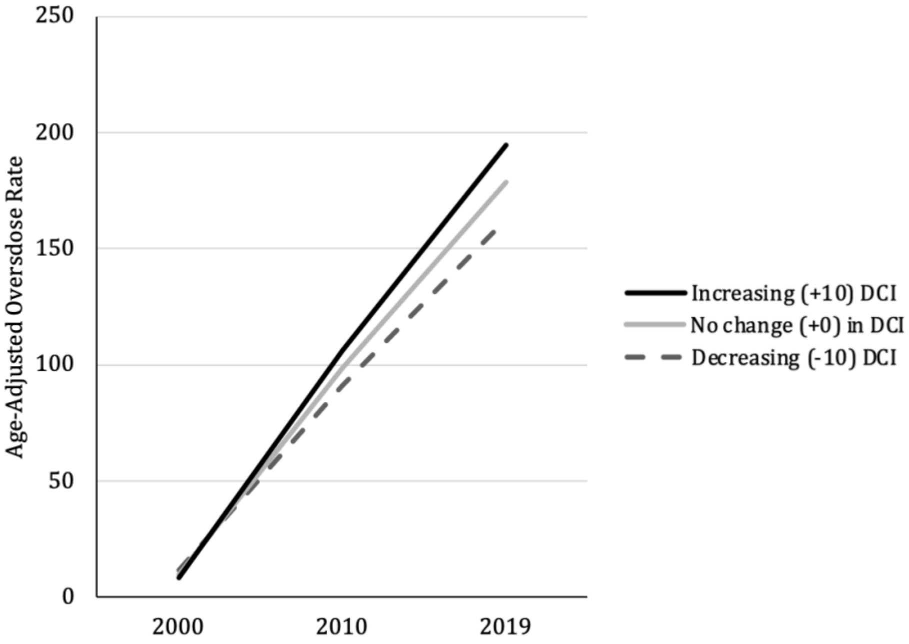

Age-Adjusted Oversdose Rate
250
200
150
100
50
0
2000
2010
2019
Increasing (+10) DCI
No change (+0) in DCI
Decreasing (-10) DCI
